# The influence of negative life events on college students’ suicidal ideation: the mediating role of entity theory and the moderating role of meaning in life

**DOI:** 10.3389/fpsyg.2024.1443474

**Published:** 2024-09-26

**Authors:** Dan Liu, Xiaowei Geng, Fuwei Zhang, Yuan Zhang, Xueli Li

**Affiliations:** ^1^College of Education, Ludong University, Yantai, China; ^2^Institute for Education and Treatment of Problematic Youth, Ludong University, Yantai, China; ^3^Jing Hengyi School of Education, Hangzhou Normal University, Hangzhou, China; ^4^School of Teacher Education (Physical Education), Taizhou University, Taizhou, China

**Keywords:** negative life events, suicidal ideation, entity theory, meaning in life, college student

## Abstract

Suicidal ideation is a desire, thought, or conception that is closely associated with suicide, which is an important risk factor for suicidal behavior. Negative life events may impact college students’ suicidal ideation. According to the suicide susceptibility-stress model, the interaction between susceptibility factors and stressors may influence college students’ suicidal ideation. The present study investigated the role of entity theory and meaning in life in the influence of negative life events on suicidal ideation among college students. A nationwide questionnaire survey was conducted among 938 college students. The Beck Scale for Suicide Ideation, the Implicit Personality Theory Questionnaire, the Adolescent Life Events Scale, and the Meaning in Life Questionnaire were used. The results showed that negative life events were positively correlated with suicidal ideation, entity theory played a mediating role, and meaning in life moderated the mediation of entity theory. Finally, meaning in life and entity theory may bring some benefits to college students; that is, when faced with negative life events, meaning in life and entity theory may attenuate students’ suicidal ideation.

## 1 Introduction

According to the *World Health Organization Yearbook*, there were more than 700,000 deaths by suicide globally in 2019. As mentioned in the report, suicide is the fourth leading cause of death for people aged 15–29 years worldwide (World Health Organization, 2019). It should be noted that nearly all college students are in this age group. In the 2022 *Chinese Health Statistics Yearbook*, suicide is listed as number 2 in the mortality data table for adolescents aged 15–25 years. Clearly, suicide not only causes loss and severe injury to one’s life and health, but also places a huge economic burden on families, schools, and society (Cerel et al., 2019). In recent years, many scholars have conducted research on suicide, investing a large amount of money each year to reduce its incidence. However, to date, the effectiveness of suicide prevention and intervention programs among college students remains unsatisfactory globally.

The majority of people experience suicidal ideation, an academic term for suicidal thoughts, before committing the act of suicide. Suicide can be seen as an ongoing process that progresses gradually from ideation to the act of carrying out the suicidal action. In the whole process of generating suicidal ideation, making a suicide plan, attempting suicide, and ultimately engaging in suicidal behavior, suicidal ideation, which is the first step, becomes the most important predictor of suicidal behavior (Feng and Xiao, 2007). One study revealed that 80% of suicidal people admitted to experiencing suicidal ideation in various forms before committing suicide (Xiao, 2001).

It has been confirmed that negative life events are a key factor in causing suicidal ideation, which plays an important role in suicidal behavior. Negative life events in the lives of college students generally include interpersonal relationship problems, freshman maladjustment, academic struggles, and emotional frustrations that lead to mental health issues. Even if the psychological development of college students is still in its early stages, they often experience confusion and may suffer from psychological barriers when faced with contradictions, challenges, and pressures in daily life. In this case, the continuous self-denial, pessimism, and disappointment among college students may even cause psychological crisis. According to Blalock et al. (2015), negative life experiences of college students not only serve as the primary cause of suicidal ideation but also act as a trigger for actual suicide. Individuals who are under prolonged pressure from negative life events are prone to negative emotions, and those who adopt negative coping styles may experience suicidal ideation and resort to suicidal behaviors (Miranda et al., 2013). Therefore, it is important to study the impact of negative life events on suicidal ideation.

Negative life experiences cause changes in individuals according to the implicit personality theory. The implicit theory of personality pertains to the belief in personality malleability. It is a fundamental cognitive schema or rudimentary theory about basic human attributes adopted by people (Yeager et al., 2013). According to the implicit personality theory, the variability in the personality of individuals is described by two types of theorists: entity theorists and incremental theorists. Entity theorists point out that an individual’s personality traits are fixed and difficult to change, while incremental theorists suggest that an individual’s personality traits are dynamic and can evolve and change (Chen and Pajares, 2010). After going through several setbacks, instead of seeking to overcome difficulties and try new approaches, many people suffer from helplessness and anxiety, which strengthens the development of an individual’s view of entity theory (Dweck, 1999). The more negative the life experiences are, the greater the learned helplessness, which leads to the negative evaluation of self-development (Dweck and Yeager, 2019). This situation can also lead to the development of an entity theory, as well as negative thoughts and behaviors.

When faced with challenges and obstacles, implicit personality theory also plays an important predictive role in the mental health of individuals (Burnette et al., 2018). It has been established that individuals described by entity theory are more likely to develop depressive states than those described by incremental theory (Kaufman et al., 2020). Students conforming to entity theory are more likely to experience negative emotions, such as sadness, and doubt their own abilities when faced with frustrating situations. On the other hand, students who adhere to incremental theory tend to have higher levels of mental fitness and mobilize all of their cognitive resources to cope positively with failures and challenges. As a result, they are less likely—perhaps never—to consider suicide (Song et al., 2022). Thus, the more negative life events they experience, the more likely they are to develop the attitudes mentioned in entity theory and to react more negatively. This belief suggests that they cannot change the bad situation, which may prompt suicidal ideation.

Therefore, H1 is proposed: *Entity theory plays a mediating role in the effect of negative life events on college students’ suicidal ideation*.

Meaning in life refers to an individual’s perception of the intention and value of life that he or she possesses in the present, as well as the pursuit of meaning and purpose in the future (Steger et al., 2008). As an important psychological resource, meaning in life is closely related to an individual’s life quality, social behavior, and physical and mental health (Czekierda et al., 2017; Kim et al., 2019). As we mentioned before, negative life events cannot be viewed without the influence of the individual’s own characteristics. Positive characteristics may play a protective role in the adaptation of some individuals, even if they have experienced bad life situations (Fergus and Zimmerman, 2005). Meaning in life also improves people’s well-being. When faced with adversity, individuals can reduce the negative effects by pursuing their life purpose, which in turn reduces the risk of developing psychological disorders (Du et al., 2017). When dealing with negative life events, entity theorists tend to make negative attributions and suggest that the environment and the self are difficult to change thereby losing expectations for the future. As a psychological resource, meaning in life will further help college students reduce suicidal ideation when negative events occur. Specifically, when meaning in life feels stronger, individuals possess more psychological resources to deal with negative events, which in turn reduces the production of suicidal ideation. High meaning in life could promote positive change, self-expansion, psychological repair, and growth (Gardner et al., 2002), enabling individuals to achieve better psychological states and avoid negative states. On the other hand, low levels of meaning in life have little effect on reducing the suicidal ideation of individuals who conform to entity theory. It is, therefore, reasonable to speculate that, for individuals with lower levels of meaning in life, the mediating role of entity theory was significant, while for those with higher levels of meaning in life, the mediating role of entity theory was not significant, as high levels of meaning in life may weaken the link between entity theory and suicidal ideation.

Therefore, H2 is proposed: *In the influence of negative life events on college students’ suicidal ideation, meaning in life moderates the mediating effect of entity theory on college students’ suicidal ideation*.

## 2 Methods

### 2.1 Participants

Using a convenience sampling method, we selected 951 college students from several cities across the eastern, central, and western regions of China. All college students voluntarily participated in the questionnaire survey. Finally, we obtained 938 valid samples, consisting of 393 males (*M* = 18.51, *SD* = 1.13) and 545 females (*M* = 19.12, *SD* = 2.06). The final number of participants was 938 because 13 questionnaires were not completely answered. The response rate of the questionnaire was 98.63%.

### 2.2 Materials

1. Adolescent Life Events Scale (ASLEC): The scale was developed by Liu et al. (1997) and consists of 27 questions covering six dimensions, including interpersonal relationships; academic stress; being punished; loss of family, friends, and property; health and adjustment problems; and other problems, for example, “heavy academic pressure.” The respondents answered on a five-point scale ranging from 1 (“no impact”) to 5 (“extreme impact”). The higher the score, the greater the impact of the event on the individual. Cronbach’s *α* coefficient of the questionnaire in this study was 0.939.
2. Entity theory was measured using the Implicit Personality Theory Questionnaire developed by Dweck (1999). The questionnaire consists of eight questions, such as, “The way a person does things can be changed; but a person’s nature, by and large, cannot be changed.” A five-point scale was used, with 1 representing “strongly disagree” and 5 representing “strongly agree.” By deducting the total score of entity theory from the total score of incremental theory, a final score is obtained. The higher the score, the more the subject favors entity theory. Cronbach’s *α* coefficient of this questionnaire in this study was 0.805.
3. The revised Beck Scale for Suicide Ideation-Chinese Version (BSI-CV) (Li et al., 2011) was used to measure suicidal ideation among college students. The 19-item scale was used to assess suicidal ideation in the past week and obtain scores for both suicidal ideation (1–5 items) and suicidal behavior tendency (6–19 items) dimensions. In the present study, questions 1–5 were used to assess suicidal ideation. The higher the score, the higher the suicidal ideation. Cronbach’s *α* coefficient for this questionnaire in this study was 0.928.
4. The Chinese Meaning in Life Questionnaire (C-MLQ) (Wang and Dai, 2008): The C-MLQ is divided into two dimensions with 10 questions in total; for example, “I understand the meaning in my life very well.” Subjects answered on a seven-point scale, with 1 representing “totally disagree” and 7 representing “totally agree” to assess their perception of meaning in life. The higher the score, the higher the level of meaning in life. The Cronbach’s *α* of the Meaning in Life Questionnaire in this study was 0.909.

### 2.3 Procedure

The current study was embedded in a larger assessment plan that analyzed the mental health status of a sample of students in different classes during orientation week. In the introductory psychology course and the mental health education course, students completed a questionnaire survey. All the students were informed of their voluntary right to take part in this study. Then, participants signed the informed consent form and completed a battery of self-report questionnaires on their smartphones, including the questionnaires mentioned above. For students who experienced psychological distress (for example, feeling depressed, anxious, or having suicidal thoughts) after completing the questionnaires, we arranged for professional counselors and psychological hotlines to address their concerns. The research team strove to ensure the mental health of every student.

### 2.4 Statistical analysis

All statistical analyses were conducted using SPSS 26.0 statistical software and PROCESS 4.1. We used Harman’s single-factor test to test for common method variance. Multiple hierarchical regression analysis was conducted to test the mediating effect of entity theory in the relationship between negative life events and suicidal ideation among college students. To further test the moderating effect, the PROCESS plug-in for SPSS 26.0 was used. In this study, statistical significance was set at *p* < 0.05.

### 2.5 Data analysis

#### 2.5.1 Common method biases

In the present research, we used Harman’s single-factor test to check the common method variance (CMV) (Aguirre-Urreta and Hu, 2019). The results showed that there were 11 factors with eigenvalues greater than 1 and the first factor explained 19.266% of the variance, which did not exceed 40%. Therefore, this study did not have a significant CMV.

#### 2.5.2 Descriptive analysis

Suicidal ideation was analyzed among college students who completed all questionnaires (*M* = 1.08, *SD* = 1.77). There were significant gender differences in suicidal ideation (*t* = −2.175, *p* < 0.05); females (*M* = 1.19, *SD* = 1.79) had significantly higher suicidal ideation than males (*M* = 0.94, *SD* = 1.73). Whether the subjects were only child had no significant effect on suicidal ideation (*t* = 0.325, *p* > 0.05). Furthermore, the subjects’ majors (*F* = 0.542, *p* > 0.05), grades (*F* = 2.231, *p* > 0.05), and age groups (*F* = 0.139, *p* > 0.05) did not significantly affect suicidal ideation (See Table 1).

**Table 1:** Difference analysis of suicidal ideation (*N* = 938).

		*M*	*SD*	*T*	*F*	*p*
1. Gender	Male	0.94	1.73	−2.175		0.030*
	Female	1.19	1.79			
2. Only child	Yes	1.11	1.82	0.325		0.746
	No	1.10	1.74			
3. Major	Science	1.05	1.72		0.542	0.582
	Liberal arts major	1.10	1.74			
	Medicine	1.28	2.30			
4. Grade	Freshman	1.08	1.75		2.231	0.083
	Sophomore	0.93	1.72			
	Junior	0.75	1.67			
	Senior	1.75	2.29			
5. Age	<20	1.10	1.79		0.139	0.870
	21–24	1.01	1.74			
	>25	1.05	1.33			

*p < 0.05.

#### 2.5.3 Correlation analysis

The means, standard deviations, and correlation coefficients for gender, age, suicidal ideation, negative life events, entity theory, and meaning in life are shown in Table 2. Suicidal ideation was positively correlated with negative life events and entity theory; suicidal ideation was negatively correlated with meaning in life. Negative life events were positively correlated with entity theory; negative life events were negatively correlated with meaning in life. Entity theory was significantly negatively correlated with meaning in life. The results are shown in Table 2.

**Table 2:** Correlation of variables (*N* = 938).

	*M*	*SD*	1	2	3	4	5	6
1. Gender	1.58	0.49	1					
2. Age	18.87	1.76	0.173**	1				
3. Suicidal ideation	1.08	1.77	0.071*	−0.13	1			
4. Negative life events	1.99	0.76	0.099**	−0.008	0.321**	1		
5. Entity theory	3.79	0.81	0.125**	0.014	0.142*	0.080*	1	
6. Meaning in life	24.11	5.55	0.075*	0.085**	−0.218**	−0.115**	−0.126**	1

*p < 0.05, **p < 0.001.

#### 2.5.4 The mediation of entity theory

**Figure 1:**
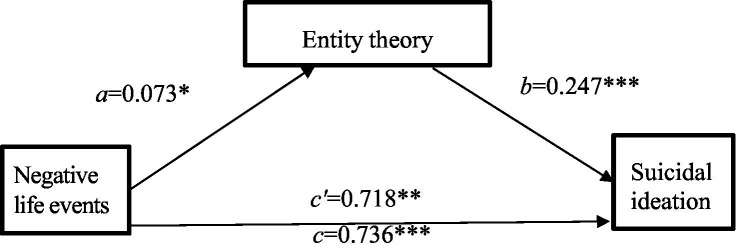
The mediation of entity theory in the influence of negative life events on suicidal ideation. *p < 0.05, **p < 0.01, ***p < 0.001.

According to Baron and Kenny (1986), multiple hierarchical regression analysis was adopted to test the mediating effect of entity theory in the relationship between negative life events and suicidal ideation among college students, as shown in Table 3. In step 1, with suicidal ideation as the dependent variable and gender, age, and negative life events as predictors, Model 1 showed that suicidal ideation was not related to gender and age (*F* = 2.669, *p* > 0.05). Model 2 showed that negative life events were a significant positive predictor of suicidal ideation (*β* = 0.736, *p* < 0.001). In step 2, with entity theory as the dependent variable and gender, age, and negative life events as the predictor variables, Model 3 showed that 1.6% of entity theory could be attributed to gender and age (*F* = 7.447, *p* < 0.01). Among these, gender was a significant positive predictor of entity theory (*β* = 0.208, *p* < 0.001), whereas age was not a significant predictor (*β* = −0.004, *p* > 0.05). Model 4 indicated that negative life events were also a significant positive predictor of entity theory (*β* = 0.073, *p* < 0.05). In step 3, with suicidal ideation as the dependent variable and gender, age, negative life events, and entity theory as the predictor variables, Model 5 indicated that 11.1% of suicidal ideation was attributable to negative life events and entity theory (*F* = 30.987, *p* < 0.001). Negative life events (*β* = 0.718, *p* < 0.001) and entity theory (*β* = 0.247, *p* < 0.001) were significant positive predictors of suicidal ideation. These results suggest that entity theory mediates the effect of negative life events on suicidal ideation among college students (see Table 3). In addition, following Preacher and Hayes’ (2008) bootstrap analysis, the mediating role of entity theory between negative life events and suicidal ideation was tested using negative life events as the independent variable, entity theory as the mediator variable, suicidal ideation as the dependent variable, and gender and age as covariates. With a generated sample size of 5,000, the indirect effect through entity theory was 0.0170, 95% CI = [0.0002, 0.0390], which illustrated the mediating role of entity theory in the effect of negative life events on college students’ suicidal ideation (see Figure 1).

**Table 3:** The mediation of entity theory in the influence of negative life events on suicidal ideation (*N* = 938).

Variables	Step1: Dependent variable: suicidal ideation		Step 2: Dependent variable: entity theory		Step 3: Dependent variable: suicide ideation
	Model 1 *β*	Model 2 *β*	Model 3 *β*	Model 4 *β*	Model 5 *β*
Gender	0.270*	0.153	0.208***	0.197***	0.105
Age	−0.026	−0.018	−0.004	−0.003	−0.017
Negative life events		0.736***		0.073*	0.718***
Entity theory					0.247***
*R*^2^	0.006	0.105	0.016	0.020	0.117
Adjusted *R*^2^	0.004	0.102	0.014	0.017	0.113
*F*	2.669	36.401***	7.447**	6.453***	30.987***

*p < 0.05, **p < 0.01, ***p < 0.001.

#### 2.5.5 The moderated effect of meaning in life

**Figure 2:**
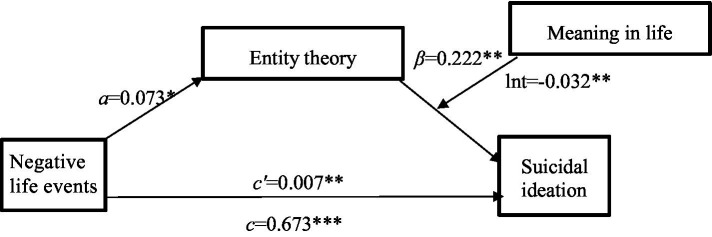
The influence of negative life events on suicidal ideation: the mediating role of entity theory and the moderating role of meaning in life. *p < 0.05, **p < 0.01, ***p < 0.001.

**Figure 3:**
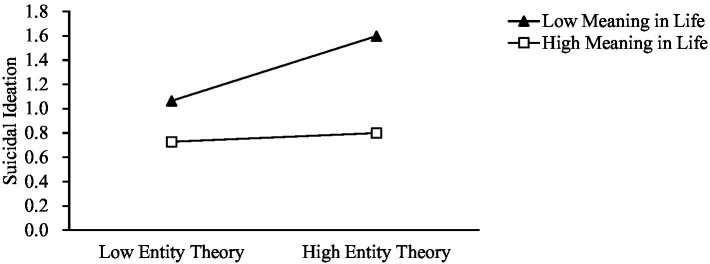
The moderation of meaning in life in the effect of entity theory on suicidal ideation.

To further test the moderating effect, a moderated mediation effect test was conducted using the PROCESS plug-in for SPSS 26.0, with reference to the bootstrap method proposed by Hayes (2013). Model 14 was selected, with a sample size of 5,000 and a 95% confidence interval, using negative life events as the independent variable X, suicidal ideation as the dependent variable Y, entity theory as the mediator variable M, meaning in life as the moderator variable V, and gender and age as the covariates. The results of the bootstrap analyses indicated that the moderated mediation model was established (see Figure 2). Specifically, meaning in life had a significant moderating effect on entity theory influencing suicidal ideation (*Effect* = −0.032, *SE* = 0.117, *p* = 0.006, 95% CI = [−0.0554, −0.0094]). Entity theory was used as a mediator variable (*Effect* = −0.002, *SE* = 0.015, 95%CI = [−0.0059, −0.0001]). In contrast, after controlling for the mediating variable (i.e., entity theory), the direct effect of the independent variable (i.e., negative life events) on the dependent variable (i.e., Suicidal Ideation) was significant (*Effect* = 0.673, *SE* = 0.071, 95% CI = [0.5336, 0.8128]), which suggested that the mediating role still held in the model. When the meaning in life scores were low (*M* − 1*SD* = −4.114), the indirect effect did not contain 0 (*Effect* = 0.026, *SE* = 0.015, 95% CI = [0.0026, 0.0588]); when meaning in life scores were high (*M* + 1*SD* = 5.386), the indirect effect contained 0 (*Effect* = 0.004, *SE* = 0.006, 95% CI = [−0.0084, 0.0160]). In other words, for subjects with low scores on meaning in life, entity theory mediated the effect of negative life events on suicidal ideation; for subjects who scored high on meaning in life, entity theory did not mediate the effect of negative life events on suicidal ideation, which suggested that meaning in life moderates the mediating role of entity theory in the influence of negative life events on suicidal ideation (see Figures 2, 3).

## 3 Discussion

This article explored the effects of negative life events, entity theory, and meaning in life on college students’ suicidal ideation. The results found that suicidal ideation was positively correlated with negative life events and entity theory; suicidal ideation was negatively correlated with meaning in life. Entity theory was significantly negatively correlated with meaning in life. Entity theory plays a partial mediating role in the influence of negative life events on college students’ suicidal ideation. Negative life events may lead college students to develop the entity view, potentially resulting in increased suicidal ideation. From the perspective of implicit personality theory, individuals who hold incremental and immutable beliefs about personality and mental health differ in the way they process information (Hong et al., 1997). Previous studies have confirmed that implicit personality theory plays an important role in mental health, and our results are consistent with previous studies that individuals conforming to entity theory are more likely to experience suicidal ideation than individuals conforming to incremental theory when faced with negative life events. Negative life events could activate the individual’s negative cognition of the self and cause the individual’s negative to engage in coping behaviors. Long-term negative life events can severely impact one’s self-esteem and self-worth. With the accumulation of negative life events, the individual’s self-evaluation continues to decrease; one may begin to doubt one’s own abilities, the surrounding environment, and others (Ma et al., 2022). In summary, negative life events can intensify one’s views as expressed by entity theory, thereby diminishing an individual’s resistance to frustration and potentially leading to psychological problems.

When faced with negative life events, the individual’s implicit personality is stimulated to play a role. When adolescents are confronted with sudden public events, those adhering to incremental theory are more likely to figure out the events positively and optimistically. Therefore, they remain low on suicidal ideation. However, individuals conforming to entity theory are more likely to act in a negative and pessimistic way, thereby prompting higher levels of suicidal ideation (Song et al., 2022). In particular, those who conform to entity theory have more negative emotions, which also accelerates the generation of suicidal thoughts, than those who adhere to incremental theory (Yang et al., 2021). Suicide is often an attempt to escape the intolerable cycle of bad events, occurring when a depressed person believes their symptoms are permanent and incurable. Furthermore, entity theory and hopelessness are highly associated (Mullarkey and Schleider, 2020). Hopelessness also leads to suicidality (Van Orden et al., 2010). When experiencing negative life events, entity theory can cause suicidal fantasies and suicidal acts, which are efforts to escape or end the psychological pain that is believed to be unchangeable. However, as a positive psychological trait, incremental theory is a protective mechanism for individuals to cope with stress, enabling them to better confront the negative events. This provides the inspiration for this study.

Meaning in life moderated the second half of the effect of entity theory on suicidal ideation among college students under the influence of negative life events. For individuals with lower scores on meaning in life, entity theory played a partial mediating role of negative life events on suicidal ideation; for individuals with higher scores on meaning in life, the mediating role of entity theory was not significant. In other words, when faced with negative life events, college students conforming to entity theory were more likely to experience suicidal ideation under the weak influence of low meaning in life scores.

Meaning in life, as a positive cognitive attribute, serves to moderate the effects of entity theory and negative life events on suicidal ideation, according to the results of this article. Individuals conforming to entity theory and facing negative events are more likely to experience suicidal ideation if they have low scores on meaning in life. This is in line with previous research confirming that a low score on meaning in life predisposes individuals to suicidal ideation, whereas a high score on meaning in life reduces it (Hu et al., 2023). Individuals with lower levels of meaning in life who are prone to feeling empty and helpless show more depression and anxiety and lower levels of well-being (Carreno et al., 2020). On the other hand, meaning in life as a positive factor can help individuals. The results of this study show that meaning in life moderates the second half of the mediation model, that is, the impact of negative life events on college students’ suicidal ideation. Specifically, for college students who conform to entity theory, the impact of negative life events on suicidal ideation gradually decreases as their sense of meaning in life increases. This result is sufficient to show that meaning in life plays an important role in suicidal ideation. On a deeper level, awareness and recognition of meaning in life can help individuals realize their potential to know what they can become and should become, so as to bring out their potential and better manifest meaning in life, always having confidence and faith to achieve goals and overcome difficulties.

## 4 Theoretical implications

The present study examined the suicidal ideation of college students based on the beliefs of an individual’s meaning in life and personality plasticity, combined with the susceptibility-stimulation model. The susceptibility stress model suggests that the interaction between susceptibility factors (personality, environment, cognition, etc.) and stress factors (negative life events, etc.) can affect suicidal ideation in adolescents. As positive qualities and protective predisposing factors, meaning in life and implicit theories enable individuals to tap into their potential and grow during stressful events. Our results enrich the susceptible stimulus model (Mann et al., 1999). Most previous research has focused on entity theory and academic performance, but the effect of implicit theory on suicidal ideation has not yet been theoretically refined. This study also confirms that entity theory has a significant impact on suicidal ideation in university students. The results expand our understanding of implicit theory.

## 5 Practical implications

This study focuses more on exploring one’s own positive attributes, with the expectation of reducing suicidal ideation. By fostering personal change, it aims to further block the occurrence of suicidal behavior. Our study has established that entity theory-conforming adolescents are more likely to experience suicidal ideation after a negative event. For the irrational cognition changing training of cognitivist therapy, we can transform those conforming to entity theory into ones following incremental theory, so that they could make better functional adaptations, recognize the specific causes of frustration, persevere in their own efforts, and seek effective strategies to solve problems (Dweck et al., 1995).

In today’s fast-paced modern society, which is full of pressures and challenges, individuals in their daily lives can easily feel pain and helplessness, resulting in a loss of life goals and a lack of value and meaning in life (Abeyta et al., 2015). This is especially true for college students, who face multiple problems such as academic obstacles and employment issues. College students with a low score of meaning in life tend to possess poor psychological resilience but a strong sense of insecurity and burnout, which forces them to cope with difficulties and challenges through more negative and passive behavioral strategies (Cho et al., 2014). This research also has significant practical implications. By offering self-growth education courses, universities can assist college students in developing a clearer sense of self-understanding and establishing their own values, ideals, and life goals. This approach can effectively improve their sense of mission and significance, which in turn will improve their sense of well-being.

## 6 Limitations and future directions

Although this study has obtained valuable results, there are still some limitations. First, the study did not collect data on psychiatric diagnosis and medication intake. Psychiatric diagnosis and drug intake may be confounding variables, which should be noted in future studies. Second, a questionnaire method was used to measure suicidal ideation, negative life events, entity theory, and meaning in life among college students. Future studies could use multiple sources of data to avoid a monolithic research approach. Additionally, this study was cross-sectional in design, lacking rigorous longitudinal comparative attribution. Causality between variables should be further explored using an experimental or follow-up study design. Finally, our study only made recommendations, lacking an intervention experiment to apply the results to confirm them. Future studies could explore the influence of implicit theory and meaning in life on college students’ suicidal ideation in more complex models. This would pave the way for establishing comprehensive intervention programs based on the influencing mechanism of suicidal ideation.

## 7 Conclusion

Suicidal ideation is positively correlated with negative life events and entity theory; suicidal ideation is negatively correlated with meaning in life. Entity theory is negatively correlated with meaning in life. Entity theory plays a partial mediating role in the influence of negative life events on college students’ suicidal ideation. In the effect of negative life events on college students’ suicidal ideation, meaning in life moderated the effect of entity theory on college students’ suicidal ideation in the second half of the model. For individuals with lower scores on meaning in life, the mediating role of entity theory was significant, while for individuals with higher scores on meaning in life, the mediating role of entity theory was not significant.

## Data Availability

The raw data supporting the conclusions of this article will be made available by the authors, without undue reservation.
